# EGFR targeting enhances the efficiency of chemotherapy through inhibiting IRE1α-XBP1s pathway in colorectal cancer cells

**DOI:** 10.7150/jca.44234

**Published:** 2020-05-18

**Authors:** Miaomiao Huo, Yahui Zhao, Xianghe Liu, Yang Gao, Die Zhang, Mengjiao Chang, Mei Liu, Ningzhi Xu, Hongxia Zhu

**Affiliations:** State Key Laboratory of Molecular Oncology, National Cancer Center/National Clinical Research Center for Cancer/Cancer Hospital, Chinese Academy of Medical Sciences and Peking Union Medical College, Beijing 100021, China.

**Keywords:** Colorectal cancer, EGFR, IRE1α-XBP1s pathway

## Abstract

Targeting EGFR combined with chemotherapy is one of the most valuable therapeutic strategies in colorectal cancer. However, resistance remains a major obstacle to improve efficacy. IRE1α-XBP1s signaling pathway is activated in many malignant tumors, and plays important roles in chemoresistance. Therefore, IRE1α-XBP1s might be a potential target to overcome the chemoresistance in colorectal cancer. In this study, we detected the activation of IRE1α-XBP1s signaling in patient cancer tissues and colorectal cancer cell lines. The phosphorylation level of IRE1α and the spliced XBP1s were aberrantly elevated in colorectal cancer, and IRE1α-XBP1s signaling activation was correlated with high EGFR expression. By overexpression of EGFR protein or activation by EGF treatment, we found that EGFR activation could enhance the phosphorylation of IRE1α and spliced XBP1s expression. On the contrary, inhibition of EGFR decreased the IRE1α-XBP1s signaling. Further, we examined the downstream signaling pathways regulated by EGFR. Inhibition of ERK activity could reverse the EGFR induced IRE1α-XBP1s activation. Co-IP confirmed the physical interaction of ERK and IRE1α. Cell growth and colony formation assay showed that the inhibition of IRE1α activity could suppress EGFR driven colorectal cancer cell proliferation. Furthermore, we found that oxaliplatin could activate IRE1α-XBP1s signaling, and combination with cetuximab partially reversed the activation. Inhibition of EGFR signaling could enhance the efficacy of oxaliplatin in vitro and in vivo. Our results showed that IRE1α RNase activity is aberrantly elevated in colorectal cancer, and EGFR signaling could activate IRE1α/XBP1s possibly through EGFR-MEK-ERK pathway. IRE1α-XBP1s pathway might involve in EGFR driven tumor cell proliferation. Cetuximab could partially recover oxaliplatin-induced IRE1α-XBP1s activation, and therefore enhance the anti-tumor efficacy of oxaliplatin. Our findings declare a new mechanism that targeting EGFR could inhibit chemotherapy-induced IRE1α-XBP1s activation and therefore enhance the efficacy.

## Introduction

Colorectal cancer is the fourth most common cancer cause of death globally. Although the prognosis of patients with colorectal cancer has improved during the past decades, still about 20% of patients with newly diagnosed colorectal cancer present with distant metastases 1. Systemic therapy remains a typical treatment option for metastatic colorectal cancer patients. A combination of chemotherapy with VEGF targeting or EGFR targeting could improve the outcome 2. However, the clinical prognosis remains unsatisfactory because of the chemoresistance 3, 4. Targeting EGFR is a valuable therapeutic strategy in metastatic colorectal cancer (mCRC), and often combined with chemotherapy. Nevertheless, treatment with cetuximab or panitumumab, is effective only in a subset of patients 5. Therefore, a better understanding of the molecular biology and therapy resistance of colorectal cancer could help to improve the therapeutic strategy.

Tumor cells are often exposed to intrinsic and external factors that alter protein homeostasis and thus induce endoplasmic reticulum stress. To cope with endoplasmic reticulum (ER) stress, the ER and nucleus communicate with each other, and the unfolded protein response (UPR) is activated 6. Inositol requiring enzyme 1 alpha (referred to as IRE1α here after, encoded by gene ern1) is one of the three major receptors in ER stress response. It is the most conserved receptor on the endoplasmic reticulum membrane 7, 8. IRE1α has both ribonuclease activity and kinase activity as reported 9. Under ER stress, IRE1α is activated 9. Activated IRE1α generates a spliced mRNA by specifically excising the 26 bp of precursor XBP1 mRNA 10, 11. The spliced XBP1 encodes an active transcription factor (XBP1s), which promotes the transcription of target genes 8. The IRE1α-XBP1s signaling pathway is activated in many malignant tumors. Compared with normal tissues, melanoma tissues have significantly enhanced the protein levels of XBP1s, and IRE1α or XBP1s overexpression could promote melanoma cell proliferation 12. The activation of IRE1α-XBP1s pathway correlates with poor patient survival in lung cancer and TNBC patients 13, 14. As a survival mechanism, IRE1α-XBP1s plays important roles in chemoresistance and radioresistance 15, 16. Studies have shown that the IRE1α-XBP1s can hinder the development of protective anti-tumor immunity by regulating the function of myeloid cells in the tumor microenvironment 17. Hence, IRE1α-XBP1s pathway can be a potential treatment target in some malignant tumors 18, 19.

XBP1s has been reported to be overexpressed in colon cancer cells, whereas it was unreactive in the normal colon epithelial cells 20.Overexpression of IRE1α or induction of XBP1s could both promote colorectal cancer cell proliferation 21, 22. But the correlation of EGFR signaling and IRE1α-XBP1s activation remains unknown. In this study, we investigate the activation of IRE1α-XBP1s signaling in colorectal cancer, and demonstrate that EGFR signaling activates IRE1α through EGFR-MEK-ERK pathway. When combined with oxaliplatin, cetuximab could inhibit the IRE1α-XBP1s activation induced by oxaliplatin and improved the therapy efficacy. These results reveal a new mechanism of EGFR targeting in the treatment of colorectal cancer.

## Materials and Methods

### Cell culture and transfection

Human colorectal cancer cell lines HCT116, HCT116 EGFR KO, SW480 were cultured in RPMI1640 supplemented with 100 U/mL penicillin and 100 μg/mL streptomycin with 10% FBS medium, and DMEM supplemented with 100 U/mL penicillin and 100 μg/mL streptomycin with 10%FBS medium was used to culture human colorectal cancer cell lines SW480 and SW620 at 37°C and 5% CO2.HCT116 EGFR KO cell line was generously provided by Dr Weina Zhang. Fetal bovine serum was purchased from ExCell Bio company, RPMI-1640 and DMEM medium were purchased from Beijing XiGong Company.

The transfection reagents used for overexpression were LipofectAMINE^TM^3000 and P3000 from Invitrogen (catalog number L3000015). pcDNA6.0-EGFR WT plasmid was provided by Mien-Chie Hung (Addgene plasmid # 42665)^23^. pcDNA3.0-HA-IRE1α was purchased from Majorbio. EGFR siRNA oligo was purchased from Cell Signaling Technology (catalog number 6480S).

### Western blot analysis

Cells of each group were harvested after treatment for 24hrs. After washing with ice-cold phosphate-buffered saline (PBS) twice, total cellular protein was prepared with RIPA lysate containing protease inhibitor and protein phosphatase inhibitor. Protein quantification was performed by BCA method, followed by SDS-PAGE gel electrophoresis. NC membrane was transferred by wet transfer method, blocked with 5% skimmed milk for one hour at room temperature, then primary antibodies (1:1000) were incubated at 4°C overnight, and HRP-labeled IgG was incubated after membrane washing. The secondary antibody (1: 2000) was incubated at room temperature for 1 h, and then exposed in a dark room. RIPA (9806S), protease inhibitor (5871S), and phosphatase inhibitor (5870S) were purchased from Cell Signaling Technology. Micro BCA™ Protein Assay Kit for protein quantitation assay was purchased from Thermo Scientific, and pre-stained protein ladder (P7712s) was from New England Biolabs. Primary antibodies were showed as follow: IRE1α (Cell Signaling Technology, 3294S); p-IRE1α (abcam, ab124945); EGFR (Cell Signaling Technology, 4277S); p-EGFR (Cell Signaling Technology, 3777S); ERK1/2 (Cell Signaling Technology, 9102S); p-ERK1/2 (Cell Signaling Technology, 9101S); XBP1 (Invitrogen, catalog number: PA5-27650); GAPDH (60004-1-Ig); Anti-mouse or anti-rabbit IgG HRP-linked secondary antibodies was purchased from OriGene.

### RNA extraction and Real-time quantitative PCR

TRizol (Catalog number 15596-026, from Invitrogen) was used to extract total RNA, then quantify and reverse transcription of mRNA was conducted. The reverse transcription reagent PrimeScript™ RT Master Mix (Perfect Real Time) was purchased from TAKARA, catalogue number (RR036A). The primers sequ-ences are listed as follow (actin-F'GCGAGAAGATGACCCAGATC, actin-R'CCAGTGGTACGGCCAGAGG; XBP1s-F'CTGAGTCCGCAGCAGGTG,XBP1s-R'TCCAAGTTGTCCAGAATGCC).Actin was used as an internal control. All reactions were performed at least 3 times and the fold change of gene expression was determined by using the 2^-ΔΔCt^ method.

### Coimmunoprecipitation assay

Immunoprecipitation kit of Proteintech^TM^ (Catalog number: KIP-2) was used to conduct coimmunoprecipitation. Cells were treated with 50ng/ml EGF for 30min before harvest and then washed twice with ice-cold PBS. According to the manufacturer's instructions, total cell lysates were incubated respectively with anti-IRE1α antibody and anti-ERK1/2 antibody overnight at 4°C. And then the protein complexes were precipitated by the addition of protein A/G agrose beads. Then the beads were washed 5 times by wash buffer. Finally, the precipitations were eluted and analyzed by Western blotting.

### Clonogenic survival assay

For clonogenic survival assay, cells were seeded in six-well plates at densities of 500 cells per well and cultured with or without EGF or inhibitors, as indicated. Two weeks later, all cells were fixed in 4% formaldehyde and stained with 0.1% crystal violet (in water).

The Epidermal growth factor (EGF) was purchased from R&D Systems Inc(catalog number 236-EG), and the IRE1α inhibitor MKC8866 was purchased from MedCheExpress (catalog number HY-104040). Both of MEK inhibitor PD032590 (catalog number S1036) and ERK SCH8477 (catalog number S7101) inhibitor were purchased from Selleck. Cetuximab was from Merck, and oxaliplatin was from Sanofi-Aventis France.

### Cell viability analysis

Cell counting kit-8 was applied to perform cell viability analysis assay. 1.5 thousand cells of SW480 each well was seeded in 96 wells plates. Cells were continuously treated with EGF (50ng/ml) or MKC8866 (10μM) after cell adherence, and cell viability was measured by CCK-8 every day for 3 days.

### Xenograft model

HCT116 cells (1×10^6^) were injected subcutaneously into male nude mice. A week later, the mice were randomly grouped and received treatments twice a week for two weeks. Nude mice bearing tumors were treated with cetuximab (25mg/kg, intraperitoneal injection), oxaliplatin (3mg/kg, intravenous injection), STF083010 (40mg/kg, intraperitoneal injection), as indicated. And mice were harvested after 4 times treatment. Tumor volumes were measured and calculated.

### Immunocytochemistry assay

This study was conducted in HCT116 cell line. Cells were cultured on coverslips in 24-well plate and treated with cetuximab (12.5μg/ml) after 24h, then immunocytochemistry assay was conducted to detect proteins of p-EGFR and p-IRE1α. After 3 times wash with PBS, precooled methanol was used for cell fixation, which treated cells for 10min. Wash twice with PBS and disrupt cell membrane by 0.2% polyoxymethylene. Then wash twice with PBS, and block with 5% BSA for one hour at room temperature. Next, primary antibodies (1:1000) were incubated at 4°C overnight. After twice wash with PBST and twice wash with PBS, HRP-labeled IgG was incubated at 37°C for 30 min. Stain with DAB, then wash with PBST and twice wash with PBS. Nuclei were stained by hematoxylin and cover the coverslips on glass slides. Primary antibodies were showed below: p-EGFR (Cell Signaling Technology, 3777S), p-IRE1α (abcam, ab48187). Secondary antibody (PV-9000), DAB (ZLI-9018) were purchased from OriGene.

### Statistical Analysis

Data in this article are presented as mean and standard error of mean. The treatment effects in each group were compared by one-way ANOVA, and differences between groups were considered significant when P<0.05.

## Results

### IRE1α RNase activity is aberrantly elevated in colorectal cancer and correlated with EGFR expression

**Figure 1:**
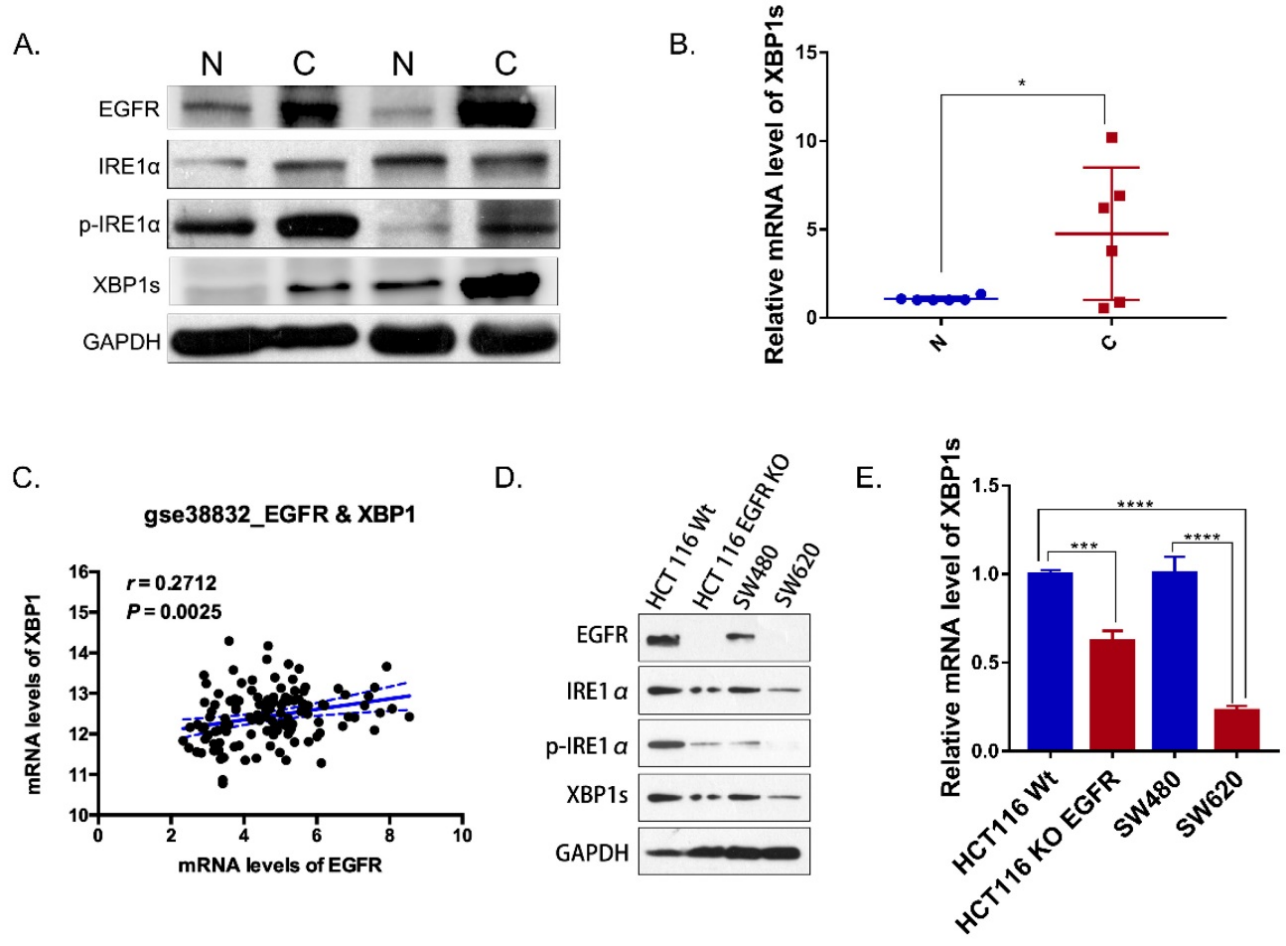
IRE1α-XBP1s pathway and EGFR expression in colorectal cancer. **A.** Immunoblotting of EGFR, IRE1α and XBP1s in paired tumor and normal tissues of colorectal cancer patients. GAPDH was used as a loading control. **B.** Expression of spliced XBP1 mRNA in 6 paired patient samples via q-PCR (n=6). Values are represented as the mean ± SD (n = 3) for each treatment (*P<0.05.) **C.** Correlation of EGFR and XBP1 in colorectal cancer patients (GSE 38832). **D.** Immunoblotting of EGFR, IRE1α and XBP1s in colorectal cancer cell lines. **E.** Expression of spliced XBP1 mRNA in colorectal cancer cell lines via q-PCR. Values are represented as the mean ± SD (n = 3) for each treatment (***P<0.001, ****P<0.0001.).

IRE1α-XBP1s signaling promotes cancer cell-intrinsic growth, metastasis and chemoresistance 7. But little is known about IRE1α-XBP1s pathway in colorectal cancer. IRE1α RNase activity was detected by evaluating the phosphorylation of IRE1α and the level of spliced XBP1. In paired tumor and normal tissues from colorectal cancer patients, total IRE1α and phosphorylated IRE1α were detected by western blot. Phosphorylation level of IRE1α in tumor tissue was higher than tumor associated normal tissue (Figure 1A). Similarly, spliced XBP1s mRNA in tumor tissue was increased in tumor tissues (Figure 1B). Also, the expression of XBP1 was correlated with EGFR in colorectal cancer patients database GSE 38822 (Figure 1C). In addition, the expression of EGFR was correlated with phosphorylated IRE1α and XBP1s (Figure 1A). In SW620 and HCT116EGFR KO cells with undetectable EGFR expression, phosphorylated IRE1α and the spliced XBP1s were lower than HCT116 and SW480 cells with high EGFR expression (Figure 1D). Spliced XBP1s mRNA expression was also increased in cells with high EGFR expression (Figure 1E). Therefore, our results indicated that IRE1α-XBP1s pathway was activated in colorectal cancer and correlated with EGFR expression.

### EGFR activation is associated with IRE1α-XBP1s Signaling

**Figure 2:**
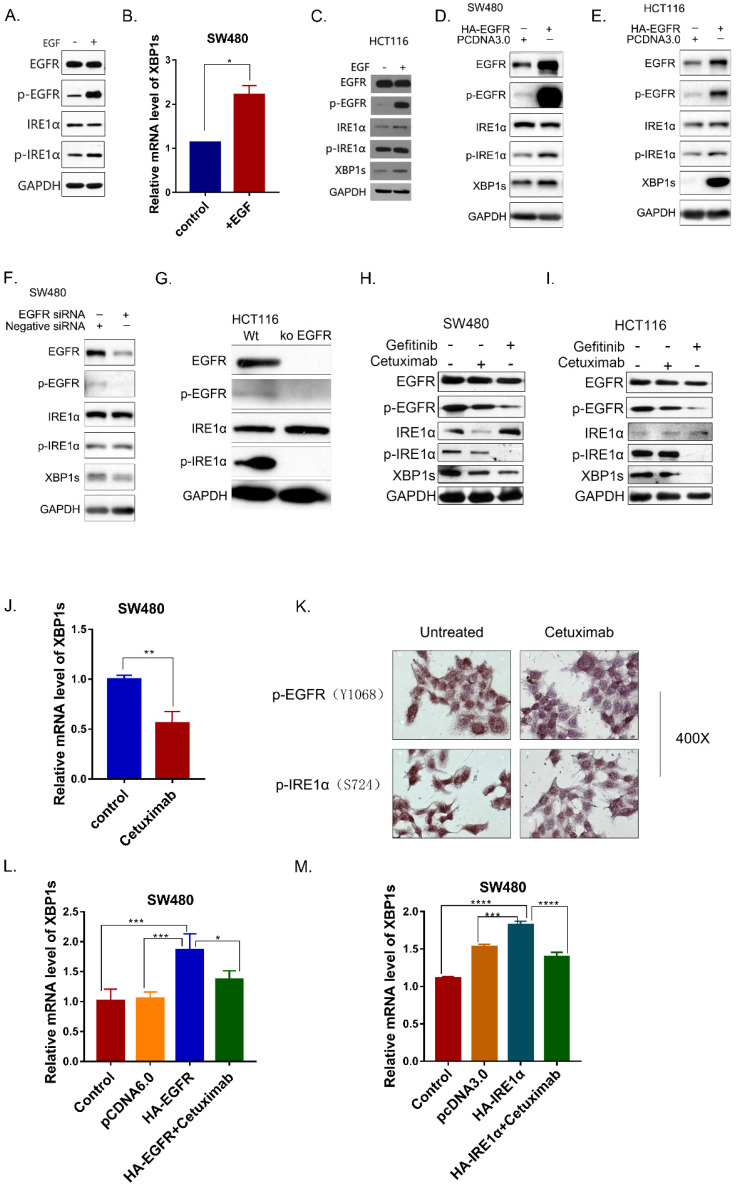
EGFR activation is associated with IRE1α -XBP1s Signaling. **A.** Phosphorylation of EGFR and IRE1α were examined in SW480 cells 30min after stimulation of EGF (50ng/ml). **B.** Expression of spliced XBP1 mRNA in SW480 cells30 min after stimulation of EGF (50ng/ml). Values are represented as the mean ± SD (n = 3) for each treatment (*P<0.05.) **C.** Phosphorylation of EGFR and IRE1α were examined in HCT116 cell line 30min after stimulation of EGF (50ng/ml) via immunoblotting. **D, E.** Activation of IRE1α-XBP1s pathway in SW480 and HCT116 were examined via immunoblotting.SW480 cells (D) and HCT116 (E) cells were transfected with pcDNA6.0-EGFR and incubated for 36h before harvest. **F.** Activation of IRE1α-XBP1s pathway was examined in SW480 cells 36h after transfection with siRNA oligos. **G.** Activation of IRE1α-XBP1s pathway was examined in HCT116 and HCT116 EGFR KO cells. **H, I.** Phosphorylation of EGFR, IRE1α and spliced XBP1 were detected in SW480 (H) and HCT116 (I) treated with cetuximab(12.5μg/ml) and Gefitinib(10nM)for 24hrs. **J.** mRNA of spliced XBP1 was detected in SW480 24 hrs after cetuximab (12.5μg/ml) treatment. Values are represented as the mean ± SD (n = 3) for each treatment (**P<0.01.) **K.** Immunocytochemistry assay was conducted to detect proteins of p-EGFR and p-IRE1α. HCT116 cells were cultured on 14mm coverslips and treated with cetuximab(12.5μg/ml) for 24hrs. **L.** Expression of spliced XBP1 mRNA was assessed in SW480 cells with different treatment. Cells were transfected with pcDNA6.0-EGFR and then treated with cetuximab(12.5μg/ml) for 24hrs before harvest. Values are represented as the mean ± SD (n = 3) for each treatment (*P<0.05, ***P<0.001) **M.** Expression of spliced XBP1 mRNA was assessed in SW480 cells, which were transfected with pcDNA3.0-HA-IRE1α and then treated with cetuximab (12.5μg/ml) for 24hrs before harvest. Values are represented as the mean ± SD (n = 3) for each treatment (***P<0.001, ****P<0.0001.)

In OSCC cells, SiEGFR inhibited IRE1α-XBP1s-GRP78 pathways 16. In addition, inhibition of EGFR could inhibit HG-induced ER stress in mesangial cell 24. To investigate whether EGFR signaling pathway is associated with IRE1α activation in colorectal cancer, SW480 cells were treated with human epidermal growth factor EGF (50ng/ml). After EGF stimulation for 30 min, EGFR was phosphorylated, and the phosphorylated IRE1α also increased (Figure 2A). As a result, the spliced XBP1s was also upregulated (Figure 2B). Similar result was obtained in HCT116 cells (Figure 2C). When EGFR was overexpressed in SW480 and HCT116 cells, phosphorylated EGFR increased, accompany by upregulation of phosphorylated IRE1α and XBP1s (Figure 2D,2E).

In order to verify this result, EGFR was knocked down by siRNA. The results showed that IRE1α-XBP1s signaling pathway was downregulated after EGFR knocked-down (Figure 2F,2G). The monoclonal antibody cetuximab and the small molecule inhibitor gefitinib were also used to inhibit EGFR activity in SW480 and HCT116 cells. Cetuximab and gefitinib both inhibited EGFR phosphorylation, and p-IRE1α and XBP1s proteins were reduced after treatment (Figure 2H,2I). Real time PCR also confirmed the downregulation of splicing XBP1s mRNA in SW480 (Figure 2J). Immunocytochemistry result also showed that cetuximab treatment reduced the phosphorylation of EGFR and IRE1α (Figure 2K). Moreover, cetuximab could decrease the XBP1s upregulation caused by EGFR overexpression (Figure 2L). We also determined whether the cetuximab could influence the spliced of XBP1 by IRE1α.We found that the overexpression of IRE1α increased XBP1s, and cetuximab could partially reverse the splicing effect (Figure 2M). Those results indicated that EGFR activation could activate the IRE1α-XBP1s signaling pathway.

### EGFR signaling activates IRE1α through EGFR-MEK-ERK pathway

**Figure 3:**
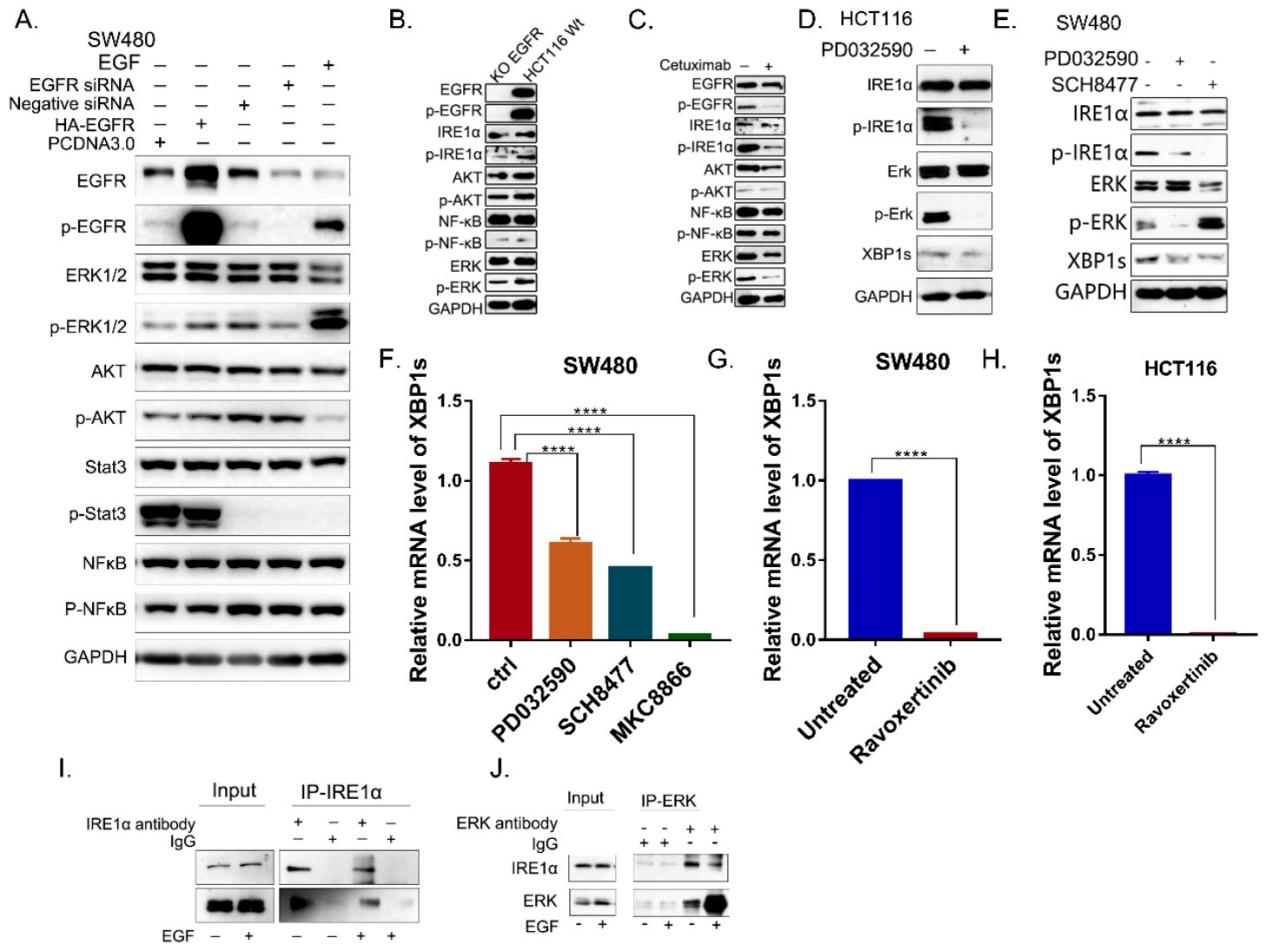
** EGFR signaling activates IRE1α through the kinase activity of ERK. A.** The molecules of EGFR pathway were detected in SW480 cells by immunoblotting. **B.** The molecules of EGFR pathway of HCT116 EGFRKO cell line and HCT116 cells were examined by immunoblotting. **C.** EGFR downstream pathway were detected by immunoblotting in HCT116 treated with cetuximab (12.5μg/ml) for 24hrs.** D.** Proteins of IRE1α-XBP1s pathway in HCT116 cells with the treatment of MEK inhibitor PD0325901 (1nM) for 24hrs were assessed by immunoblotting. **E.** p-IRE1α (S724) and XBP1s were assessed via immunoblotting after the treatment of MEK inhibitor PD0325901 (1nM) or ERK inhibitor SCH8477 (5μM) for 24hrs. **F.** Expression of spliced XBP1 mRNA was assessed in SW480 cells after treatment with MEK inhibitor PD0325901 (1nM) or ERK inhibitor SCH8477 (5μM). MKC8866 was used as positive control. Values are represented as the mean ± SD (n = 3) for each treatment (****P<0.0001.) **G, H.** Expression of spliced XBP1 mRNA was assessed in SW480 (G) and HCT116 (H) cells after treatment with ERK inhibitor Ravoxertinib(10nM)for 24hrs. Values are represented as the mean ± SD (n = 3) for each treatment (****P<0.0001.) **I, J.** Coimmunoprecipitation was conducted in SW480 cell line.

To elucidate the mechanism by which EGFR signaling regulates IRE1α activity, the downstream signaling pathways activated by EGFR were examined. As shown in Figure 3A, only the phosphorylation of ERK was positively correlated with the activation of EGFR in SW480 cells. When EGFR was knocked-down, p-EGFR, p-ERK and p-IRE1α were significantly reduced in HCT116 (Figure 3B), and there is also this phenomenon when treated with cetuximab, in HCT116 cell line (Figure 3C). Then the inhibitor of MEK, PD0325901 was use to inhibit the activation of ERK. With the treatment of PD0325901, the phosphorylation level of ERK decreased, and HCT116 cells showed IRE1α-XBP1s signaling pathway downregulation (Figure 3D). Also, both MEK inhibitor PD0325901and ERK Inhibitor SCH8477 could effectively inhibit the phosphorylation of IRE1α and XBP1s protein level (Figure 3E,3F). And when treated with Ravoxertinib, another inhibitor of ERK, the splicing XBP1s was also significantly inhibited in HCT116 and SW480 cells (Figure 3G,3H). Further CO-IP experiment showed that ERK and IRE1α were physically interacted (Figure 3I,3J). Altogether, these results suggested that EGFR signaling could regulates IRE1α activity possibly through EGFR-MEK-ERK pathway.

### Inhibition of IRE1α-XBP1s pathway suppresses EGFR driven tumor cell proliferation

**Figure 4:**
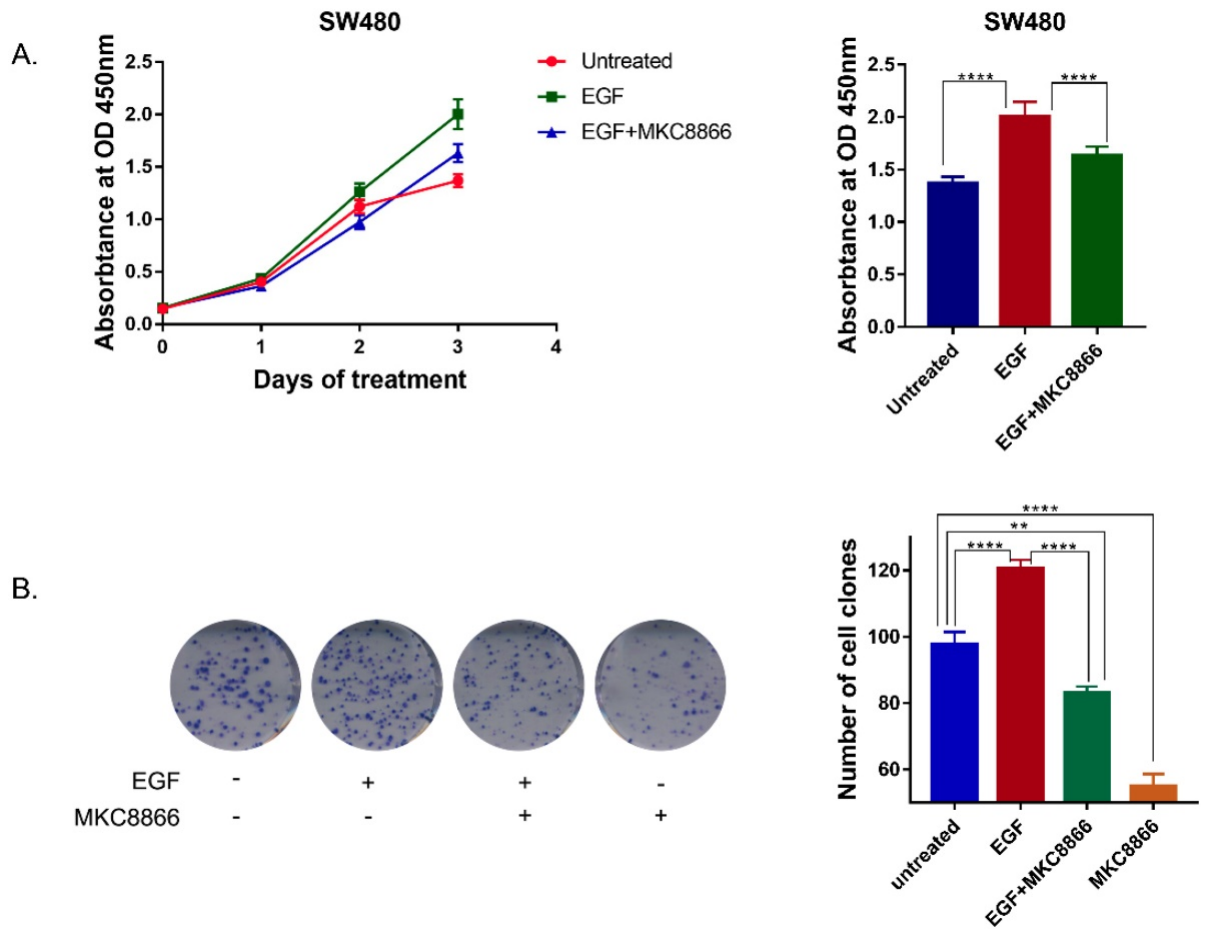
** Inhibition of IRE1α-XBP1s pathway suppresses EGF-induced cell proliferation. A.** Growth curve of SW480 cells. 1.5 thousand cells of SW480 each well was seeded in 96 wells plates. Cells were treated with EGF (50ng/ml) or MKC8866 (10μM), and cell proliferation was monitored by CCK-8 assay every 3 days. Values are represented as the mean ± SD (n = 10) for each treatment (****P<0.0001.) **B.** Colony formation result of SW480 cells.0.5 thousand cells of SW480 each well were seeded in 6 wells plates. Cells were treated with EGF (50ng/ml) or MKC8866(10μM) and 12 days later cells were stained with 0.1% crystal violet (in water).Values are represented as the mean ± SD (n = 3) for each treatment (***P<0.001, ****P<0.0001.)

In order to verify the effect of IRE1α-XBP1s on EGFR driven cell proliferation, IRE1α RNase activity was blocked by MKC8866, an IRE1α RNase inhibitor. With a sustaining stimulation of EGF (50ng/ml), the proliferation rate of SW480 cells accelerated, and treatment with MKC8866 (10μM) decreased the proliferation rate (Figure 4A). The clonogenic survival assay, also showed that MKC8866 could effectively inhibit colony formation of SW480 cells (Figure 4B). The result indicated that IRE1α-XBP1s pathway might participate in EGFR driven tumor cell proliferation.

### Inhibition of EGFR signaling enhances the effectiveness of oxaliplatin via inhibition of IRE1α-XBP1s pathway

**Figure 5:**
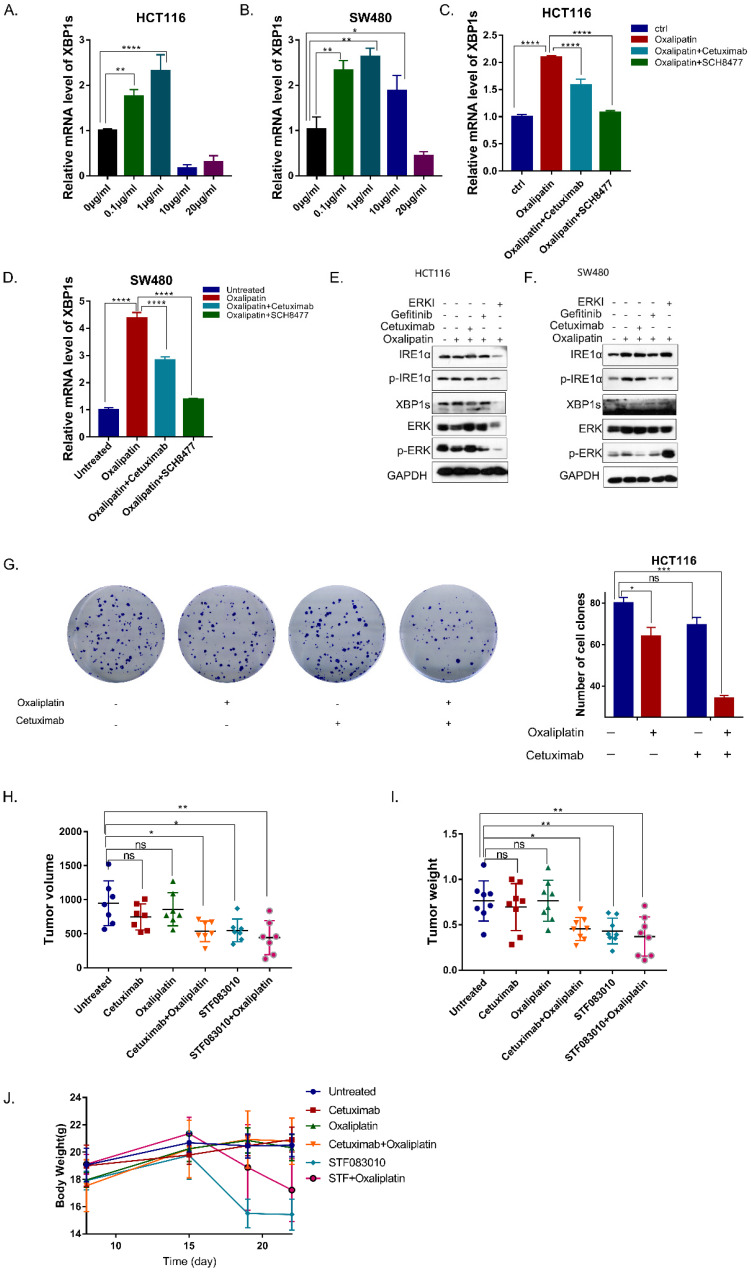
** Cetuximab enhances the efficacy of Oxaliplatin by decreasing the activation of IRE1α-XBP1s. A, B.** XBP1s expression was detected 24 hrs after oxaliplatin treatment in HCT116 (A) and SW480 (B) cells. Values are represented as the mean ± SD (n = 3) for each treatment (**P<0.01, ****P<0.0001.) **C, D.** XBP1s expression was detected in HCT116 (C) and SW480 (D) cells treated with 1μg/ml oxaliplatin combined with cetuximab or ERK inhibitor SCH8477 for 24hrs. Values are represented as the mean ± SD (n = 3) for each treatment (****P<0.0001.) **E, F.** Immunoblotting detection for p-IRE1α and XBP1s. GAPDH was used as a loading control. **G** Colony formation result of HCT116 cells.0.5 thousand cells of HCT116 each well were seeded in 6 wells plates. Cells were treated with oxaliplatin (1μg/ml) or cetuximab (12.5μg/ml) for12 days, and stained with 0.1% crystal violet. Values are represented as the mean ± SD (n = 2) for each treatment (ns, P>0.05, *P<0.05, ***P<0.001). **H, I.** In vivo tumor growth analysis. Nude mice were subcutaneously injected with HCT116. Nude mice bearing tumors were treated with cetuximab (25mg/kg, intraperitoneal injection), Oxaliplatin (3mg/kg, intravenous injection), STF083010 (40mg/kg, intraperitoneal injection), as indicated. Two weeks later, mice were sacrificed. Tumor volume (H) and tumor weight (I) were shown. Values are represented as the mean ± SD from seven mice per group. (ns, P>0.05,*P<0.05,**P<0.01). **J.** Body weight changes of nude mice after treatment.

As an important survival mechanism of cells, UPR response plays an important role in the resistance of tumor cells to chemotherapy, which often causes chemoresistance due to the activation of IRE1α, leading to poor prognosis^15^.To determine whether oxaliplatin could activate IRE1α RNase activity, HCT116 and SW480 cells were treated with oxaliplatin of different concentrations. Low dose oxaliplatin increased IRE1α RNase activity as demonstrated by an increase in levels of spliced XBP1s (Figure 5A,5B). Moreover, addition of MKC8866 was sufficient to completely block oxaliplatin-induced expression of XBP1s. Further, combination of cetuximab or ERK inhibitors with oxaliplatin could suppress the splice level of XBP1 (Figure 5C,5D). Western Blot result also showed that the phosphorylation level of IRE1α and XBP1s were upregulated by oxaliplatin and inhibited by EGFR inhibitor or ERK inhibitor (Figure 5E,5F). Collectively, cetuximab can decreased the ERS caused by oxaliplatin. Clonogenic survival assay showed that the combination of cetuximab could enhance the effectiveness of oxaliplatin (Figure 5G). To test the effects of the chemosensitivity in vivo, HCT116 cells were subcutaneously injected into nude mice. As shown in Figure 5H, tumor volumes and tumor weights were significantly inhibited in the group treated with oxaliplatin in combination with cetuximab, STF083010, an inhibitor of IRE1α (Figure 5H,5I). But significant body weight loss was only observed in the mice treated with STF083010 (Figure 5J). Taken together, Inhibition of EGFR signaling could enhance the effectiveness of oxaliplatin via inhibition of IRE1α-XBP1s pathway.

## Discussion

Due to nutritional deficiencies, hypoxia, high metabolic requirements, and oxidative stress in tumors, the ER folding function is disrupted and ER stress is generated. ER stress and UPR activation are documented in the development of many cancer types 25. IRE1α-XBP1s pathway is activated in many kinds of cancers, but there are few reports on the status of IRE1α-XBP1s in colon cancer. In colorectal cancer XBP1s mRNA has been reported to be overexpressed in colon cancer cells 20. Immunohistochemical staining result showed the high expression of total IRE1α indicated shorter overall survival time of CRC patients 22. In this study, we detected the phosphorylation of IRE1α and found the phosphorylation level of IRE1α in tumor tissue was higher than tumor associated normal tissue.

The expression level of spliced form XBP1 (XBP1s) showed the similar pattern. The result indicated that not only the overexpression but also the activation of IRE1α-XBP1s pathway existed in colorectal cancer. Previous reports showed that activation of IRE1α-XBP1s in colorectal cancer cells could promote cell proliferation 21, 22. As a transcriptional factor, XBP1s could promote tumor cell proliferation through its target genes. XBP1s could directly regulate the expression of Twist, mediating EMT in HCC cells and the invasion and metastasis of HCC 26. IRE1α-XBP1s promoted melanoma cell proliferation by directly regulating IL-6 transcription 12.XBP1s could directly activate c-MYC expression 27. Therefore, inhibition of IRE1α-XBP1s signaling might be a potential target in the treatment of colorectal cancer.

Our results also indicated that IRE1α-XBP1s activation was correlated with EGFR expression in colorectal cancer. Some previous results also confirmed the correlation. EGF could rapidly increase splicing of XBP1s mRNA in breast cancer cells, and pre-treating cells with the EGFR inhibitor, erlotinib, blocked EGF-induction of spliced XBP1 mRNA 28. In OSCC cell lines, EGFR silencing reduced the phospho-IRE1α levels as well as splicing of XBP1s 16. Then, we explored the detailed mechanism. EGFR activates the downstream kinase cascade and promotes colorectal cancer progression through the increased cell proliferation, prolonged survival, angiogenesis, anti-apoptosis, invasion, and metastasis 29. Previous reports have showed the activation of ERK was necessary for IRE1α-XBP1s activation. Inhibition of MEK or knockdown of ERK1/2 could prevent tunicamycin-induced IRE1α activation 30, 31. We found that EGFR signaling activated IRE1α through EGFR-MEK-ERK pathway, inhibition of MEK or ERK could reduce the IRE1α phosphorylation and XBP1s expression in colorectal cancer cell lines. We have proved a physical interaction between ERK and IRE1α through coimmunoprecipitation, and ERK might directly phosphorylate IRE1α upon EGFR activation. Furthermore, when IRE1α RNase activity was blocked by MKC8866, EGFR driving cell proliferation was inhibited. Taken together, EGFR signaling might promote cancer progression partially through activation of IRE1α-XBP1s pathway.

Inhibition of EGFR is usually combined with other chemotherapy in colorectal cancer treatment. Oxaliplatin and irinotecan combined the EGFR antibodies cetuximab and pemetrexumab are widely used clinically 32. Oxaliplatin is used for treatment of colorectal cancer, especially for metastatic colorectal cancer 33. But intrinsic or acquired resistance still is the major cause of treatment failure. The molecular mechanism of oxaliplatin resistance includes alterations in transport, detoxification, DNA damage response and repair, cell death and epigenetic regulations 34. Understanding the molecular mechanisms helps to find ways of circumventing it and to improve and optimize treatments. Oxaliplatin could induce ER stress. Targeting oxaliplatin induced UPR might be a potential strategy. Oxaliplatin induced autophagy and protect against oxaliplatin-induced cell death, but blocking ER stress by RNA interference could decrease autophagy and enhance the oxaliplatin induced cell death 35. In this study, oxaliplatin could induce IRE1α-XBP1s pathway activation. As a survival mechanism, IRE1α-XBP1s signaling could promote colorectal cancer cell proliferation 21, 22. Therefore, activation of IRE1α-XBP1s pathway might be a big obstacle for oxaliplatin to inhibit tumor growth. When combined with EGFR targeting, ERK inhibitor or IRE1α inhibitor, the IRE1α-XBP1s activated by oxaliplatin could be reduced, and *in vivo* and *in vitro* experiments both showed the enhanced inhibition effectiveness of oxaliplatin.

In summary, we demonstrated that targeting EGFR could inhibit the activation of IRE1α-XBP1s signaling through EGFR-MEK-ERK pathway. Combined with cetuximab could enhance the effectiveness of oxaliplatin via inhibition of IRE1α-XBP1s pathway. Our findings indicate IRE1α-XBP1s signaling is a potential therapeutic target and provides strong evidence to support the use of EGFR targeting in combination with chemotherapeutics for the treatment of colorectal cancer.
